# Support pressure distribution for positioning in neutral versus conventional positioning in the prevention of decubitus ulcers: a pilot study in healthy participants

**DOI:** 10.1186/s12912-017-0253-z

**Published:** 2017-10-16

**Authors:** Heidrun Pickenbrock, Vera U. Ludwig, Antonia Zapf

**Affiliations:** 1 St. Mauritius Therapieklinik, Bereich Motorik, Strümper Straße 111, 40670 Meerbusch, Germany; 2 Berlin School of Mind and Brain, Humboldt Universität zu Berlin, Berlin, Germany; 3 Department of Medical Statistics, University Medical Center Göttingen, Göttingen, Germany

**Keywords:** Positioning, Pressure measurement, Decubitus, Positioning in neutral, Pressure distribution

## Abstract

**Background:**

Decubitus ulcers are associated with a burden for the patients and cause enormous costs. One of the reasons for the development of decubitus is prolonged exposure to pressure. The aim of this pilot study was to examine the pressure distribution of healthy individuals either positioned in Positioning in Neutral (LiN) or conventional positioning (CON).

**Methods:**

Four healthy participants were positioned in a supine, 30° degree side lying and 90° side lying position both in LiN and CON. A thousand pressure sensors in a mattress enabled a visual presentation of low, medium and high pressure on a screen. This presentation was processed by Photoshop in order to count the pixels representing the total support pressure surface and the pressure intensity.

**Results:**

LiN showed, on average, a smaller surface with measurable pressure compared to CON (46,293 versus 64,090 pixels). The areas of medium pressure were comparable. Mean areas of low and high pressure were both smaller in LiN as compared to CON (low: 8315 versus 22,790 pixels; high: 3744 versus 7277 pixels).

**Conclusion:**

The results of this pilot study indicate that LiN is suitable for pressure sore prophylaxis because LiN showed less support surface and less maximum pressure as compared to CON.

## Background

Immobile patients are at constant risk for developing decubitus ulcers. This is particularly true for patients with geriatric disorders. The exact incidence and prevalence is unknown, but it is estimated that 400,000 people in Germany suffer from a decubitus that is in need of treatment. The prevalence in German hospitals has been estimated to be about 10% in geriatric clinics, 30% in retirement homes and 20% for patients cared for in a home environment [1]. In stationary health care, patients with the highest risk are those with a higher age, longer duration of the stay, treatment at the intensive care unit and transfer from a stationary care facility [2]. Mean estimated costs for therapy of a decubitus ulcer are 50,000 € [3]. In Germany, treatment costs, prolonged stays at the hospital and inability to work due to the condition are estimated to produce an economic damage of about 1 to 2 billion € per year. Severens et al. estimate that in 2010, the costs correspond to 1% of the entire health budget of the Netherlands [4]. Thus, besides the pain and long duration of the healing process of such an injury for each individual patient, decubitus ulcers also play a large economic role [5].

A main reason for developing pressure sores, besides immobility and malnutrition, is believed to be sustained pressure on the skin as well as pressure combined with shearing force [1, 6]. The pressure leads to a compression of the blood vessels, reduction of oxygen perfusion with local ischemia and, as a consequence, the formation of necrosis [7, 8]. To avoid sustained pressure, patients at risk have to regularly be put into different positions. In case that this is insufficient, the patient is positioned on antidecubitus soft foam or alternating pressure mattresses, which reduce the total pressure [9]. The disadvantage of these mattresses, however, is that they compromise patients’ perception of the body. Moreover, they hinder patients’ activities and impede nursing care [3]. In a European prevalence study, it was shown that only 10% of patients at risk for developing decubitus ulcers receive adequate preventive treatment [10].

**Fig. 1:**
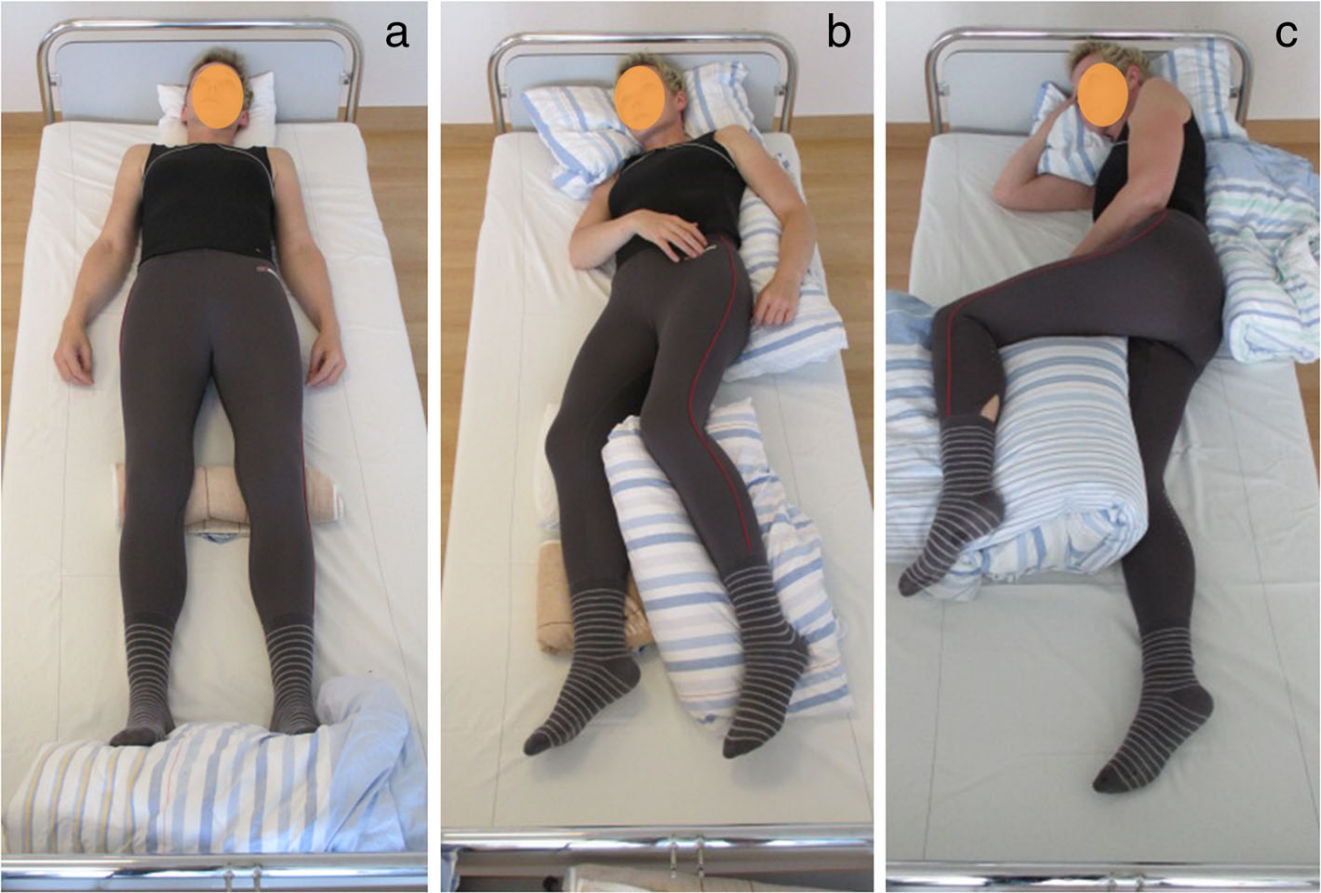
A healthy woman positioned in those CON positons which were used in this study **a**) supine, **b**) 30° side lying and **c**) 90° side lying

Positioning is performed by turning patients into different positions such as supine, 30° side lying, 90° side lying or prone lying. Conventionally, during positioning nurses focus on placing support materials at specific parts of the body (e.g., at the back, under the leg). Due to gravity the body adapts to the mattress, and the effect this has on the alignment of the body parts is accepted. Hollow spaces can occur. Support material is intended to be used quite sparingly (Fig. 1).

A possible alternative solution is Positioning in Neutral (*Lagerung in Neutralstellung, LiN*), a positioning concept developed a few years ago. Initially, this method was developed for patients with central nervous system damage; nowadays it is increasingly being used for immobile patients with other symptoms and disorders.

Attention to the alignment of body parts is the basic principle of the LiN approach. The joints are positioned as neutrally as possible. A joint is in neutral when it is not flexed, extended, abducted, adducted or rotated. The purpose is to avoid overstretching and shortening of muscles. For example, in supine-, prone- and 30° side lying the posture looks like a person standing upright as long as the patient does not have contractures. Turned 90° to the side one or both legs are flexed but abduction, adduction and rotation are avoided. All parts of the body are supported against the influence of gravity. Paretic body segments, regardless of high or low tone, are stabilized with special techniques to normalize tone. Hollow spaces should be avoided and therefore filled. A sufficient number of blankets and pillows is needed to follow these principles. As a result, the material offers a much broader base of support than CON. The weight of the body is distributed equally. Areas of high risk to develop decubitus ulcers such as heels, ischium and sacrum are exposed to less pressure compared to CON. Each conventionally used position can be converted from CON to LiN.

**Fig. 2:**
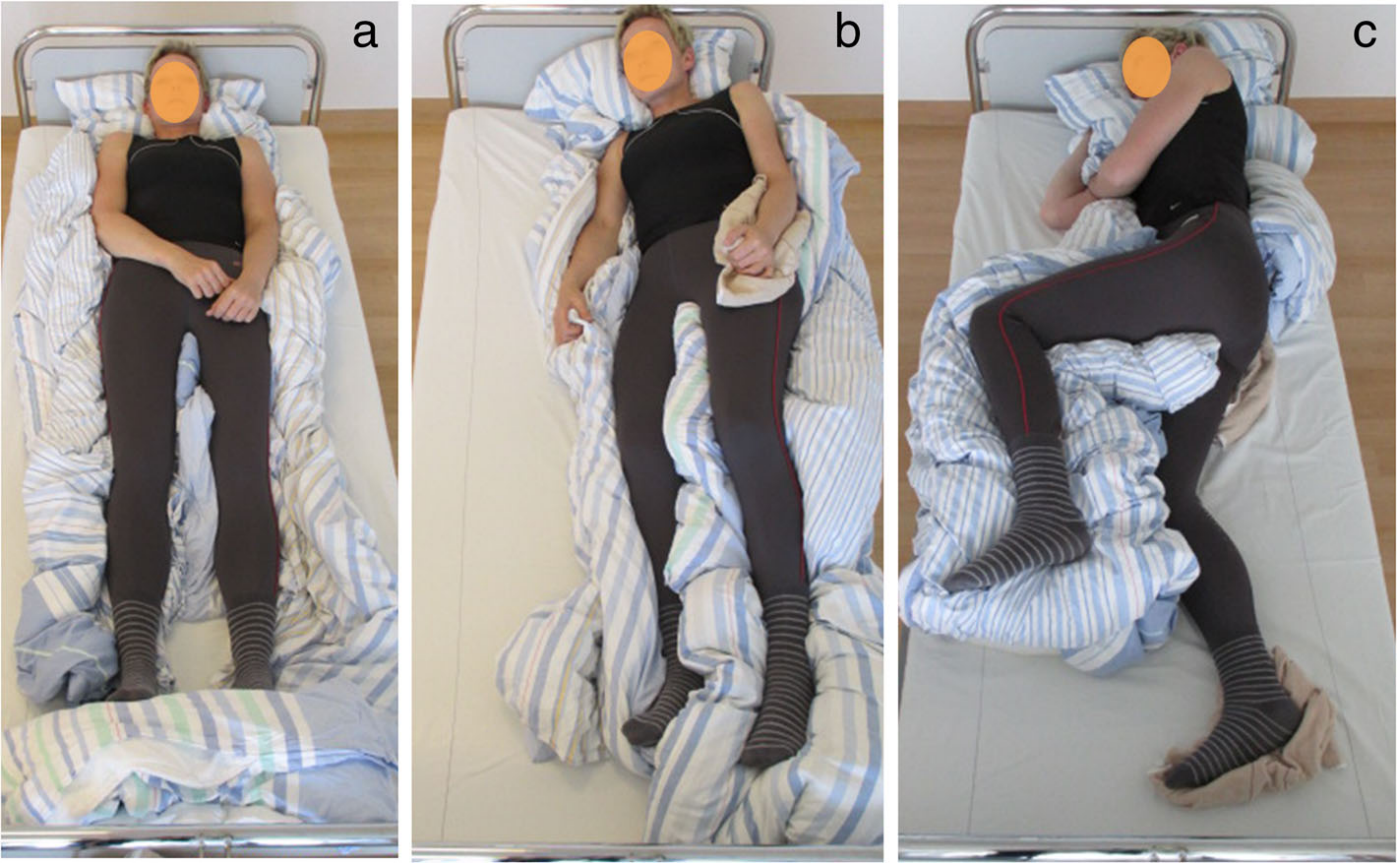
A healthy woman positioned in those LiN positons which were used in this study: **a**) supine, **b**) 30° side lying and **c**) 90° side lying

In a multicentre randomized controlled trial, it was shown that LiN lead to improved passive mobility of patients’ hips and shoulders compared to conventional positioning (CON). Furthermore, LiN was perceived as substantially more comfortable than CON [11]. Neither LiN nor CON changed patients’ pulse, blood pressure or respiratory rate [12]. In LiN, all body parts are brought, as much as possible, into a neutral zero-position and are stabilized using special techniques, so that they do not malalign due to gravity. For this purpose, more positioning material is needed compared to CON. Body parts are evenly supported without allowing empty spaces. In LiN, there is a larger total surface area on which the patient’s weight is distributed compared to CON (Fig. 1). The aim of this pilot study was to investigate whether LiN, compared to CON, leads to a lower total support pressure and in particular to smaller areas of high pressure (Fig. 2).

## Methods

**Fig. 3:**
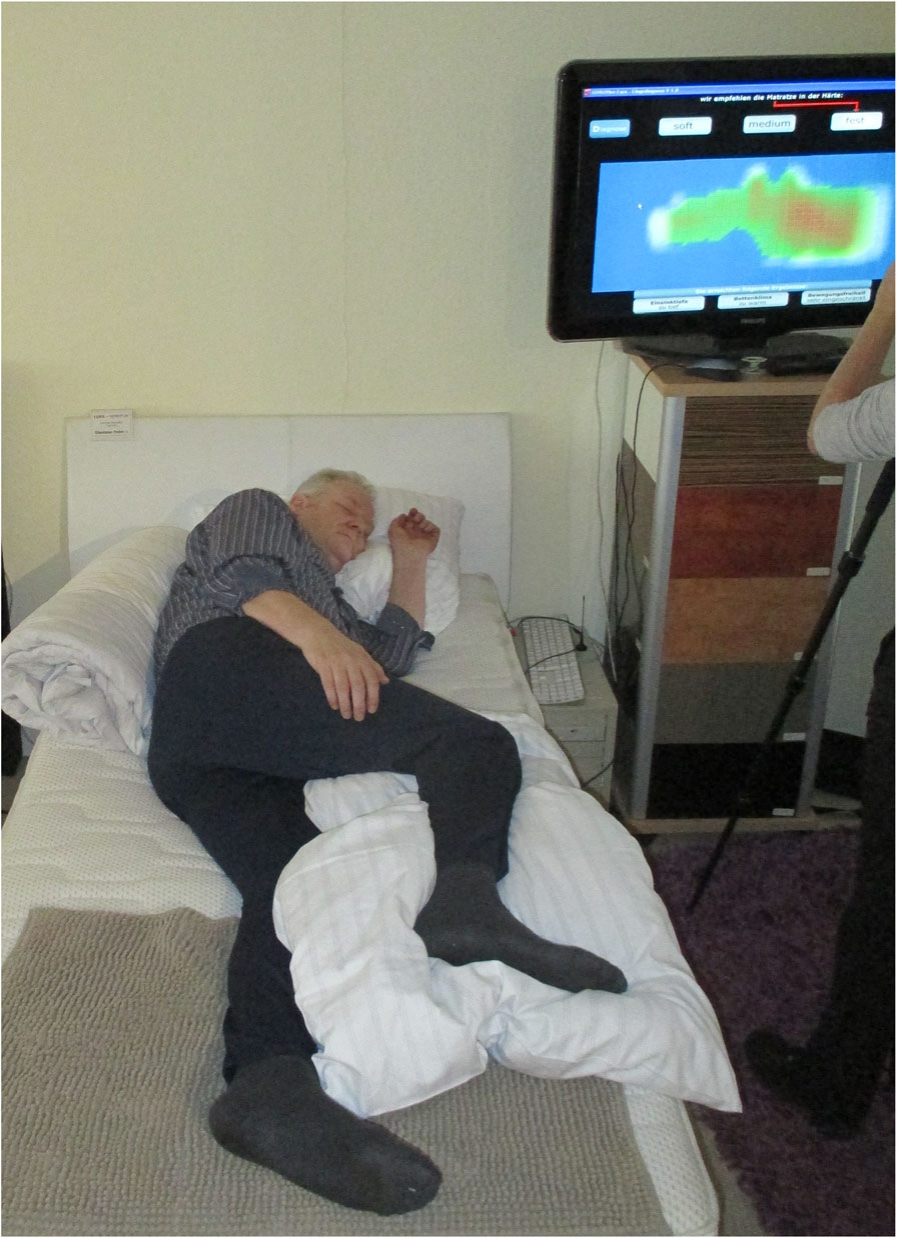
Picture of the set-up. Participant with pre-obesity in a 90° side lying position (CON), showing the measurement of the pressure distribution on the monitor on the right

**Fig. 4:**
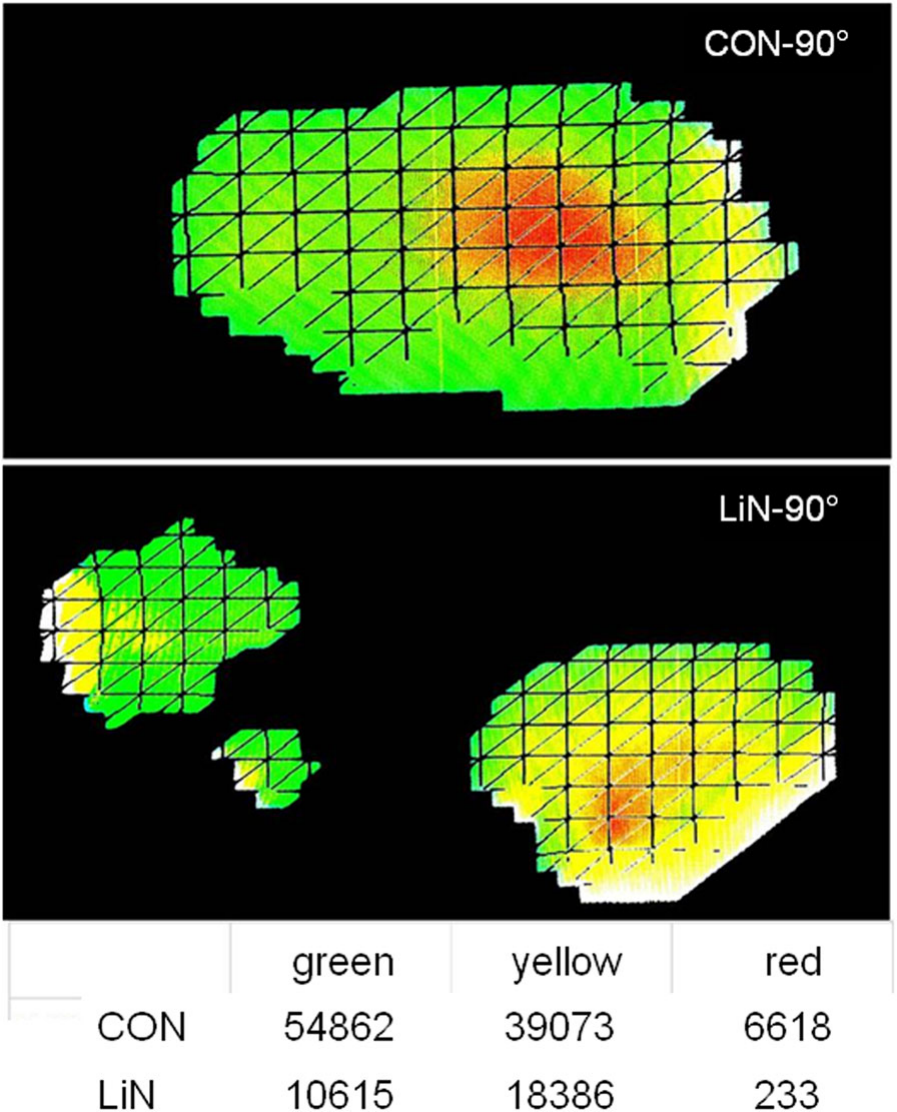
Pressure measurement of a participant in 90° side lying. Image was processed in Photoshop so that only areas with low pressure (green pixels), medium pressure (yellow pixels) and high pressure (red pixels) are shown. Areas with no pressure are not shown. The table underneath shows the total number of pixels per category

Four healthy participants were positioned in three different positions each in both LiN and CON: supine, 30° side lying (left or right), 90°side lying (left or right). These are the commonly used positions for patients in need for positioning. The order of positions was randomized. For this purpose, each participant drew two concept cards (LiN, CON) and three out of five position cards (supine, 30° side lying left and right, 90° side lying left and right) from a pile until both concepts were drawn three times each and all three types of positions were used. For 30° side lying and 90° side lying the side of the body (left or right) was chosen that was drawn first, and if the position was drawn again for the same concept and the other side (left or right), it was discarded and the next card was drawn. The order of the conditions was documented. Pictures of all positions were taken (Figs. 3 and 4).

Participants were two men (age 54 and 59 years) and two women (age 42 and 56 years). One man and one woman were in the normal weight range with a body mass index (BMI) of 20.8 and 21 respectively. One man was in the pre-obesity range (BMI: 26.8), and one woman was obese (BMI 36.7).

A nurse experienced in both concepts performed all positioning. The positions were carried out as in Pickenbock et al. [11]: For LiN, the original instruction sheets of the concept were used. For CON, instructions were derived from the literature [11]. The respective worksheets can be obtained by mailing the corresponding author.

Data collection took place in December 2015 in a large store for mattresses in Krefeld, Germany. This store has at its disposal a pressure measurement device that allows customers to find out about their individual pressure zones while lying on a mattress. On a screen, the visualization of a thousand sensors in the mattress shows the pressure that results when a person is lying on the pressure mattress. Differential pressure is marked by the colours green for low, yellow for medium and red for high pressure. Areas without pressure appear in blue (Fig. 2). This means that the system cannot reveal the overall area of pressure, but only those areas with a certain minimum pressure. Besides this, the pressure measurement system generates recommendations for lying positions and mattresses which were not taken into account for the analysis in this study. The visualization of the pressure distribution in each position was captured by a digital camera, which was fixated on a tripod, to ensure equal conditions for each picture.

Pictures were processed using Adobe Photoshop CS6–64 Bit. First, we selected the area of the screen which only showed green, yellow and red areas. Then, all remaining shades of blue were erased from the peripheral areas of the picture, by setting hue and saturation of blue and cyan to a value of 0. The contrast of the remaining picture was maximized. With Photoshop’s “count pixel” function for the selection of green, yellow and red, the number of pixels of each colour category were determined (Fig. 3).

**Fig. 5:**
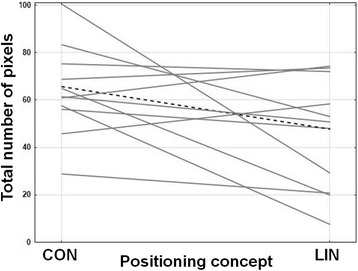
Profile plot for all 12 measurements per positioning concept. Each line corresponds to one participant and to one type of position (supine, 30° or 90° side lying) in both concepts. Note that the three measurements per participants are dependent. The black line represents the mean value over the 12 value pairs

Since the study is explorative and hypotheses-generating and since the sample size is small, the data were analyzed descriptively. As statistical measures the arithmetic means of the absolute number of pixels in the red, green, and yellow areas were calculated over the four participants, separately for the three positions (supine, 30° and 90° side lying*)* and the two positioning concepts (LiN and CON). In addition, the absolute total number of pixels of all measurements (three positions for four participants, that is 12 measurements per positioning concept) were graphically compared between LiN and CON positioning using a profile plot. Note that the measurements regarding the same participant are dependent (Fig. 5).

## Results

The mean area with measureable pressure over all 12 measurements (i.e., four participants with three positions each) was smaller in LiN (46,293 pixels) compared to CON (64,090 pixels). Note that the actual support surface is theoretically the same for both positioning concepts, because participants were the same in the LiN and the CON condition. Thus, the large difference of 17,797 pixels on average means that with LiN positioning, measureable pressure is exerted on a surface that is 28% smaller compared to CON. While the surface with medium pressure (yellow) was comparable for both positioning concepts (LiN 212 pixels more than CON), the surfaces with low (green) and high (red) pressure were relevantly smaller for LiN than for CON (green: 14,475 pixels less; red: 3533 pixels less). Details are provided in Table 1.

**Table 1:** Mean number of coloured pixels for both concepts separately and for the difference (CON-LiN)

		All positions	Supine	30° SL	90° SL
Sum	CON	64,090	59,853	50,401	82,014
	LiN	46,293	37,477	44,406	56,996
	Difference	17,797	22,376	5995	25,017
Low pressure (green)	CON	22,790	32,285	11,189	24,894
	LiN	8315	5782	7981	11,180
	Difference	14,475	26,503	3207	13,714
Medium pressure (yellow)	CON	34,023	21,541	31,000	49,526
	LiN	34,235	29,319	33,856	39,529
	Difference	−212	−7777	−2855	9997
High pressure (red)	CON	7277	6025	8212	7593
	LiN	3744	2375	2568	6287
	Difference	3533	3650	5643	1305

*N* = 4. SL: side lying. CON: conventional positioning. LiN: Positioning in Neutral (Lagerung in Neutralstellung)

For nine of the 12 paired measurements (i.e., LiN and CON for the same participant and the same position) the total support surface with a measurable pressure was smaller for LiN than for CON. For the other three paired measurements (where the total surface with a measureable pressure was larger for LiN) the surface with high pressure (red) was smaller for LiN or, for one participant, equal for both concepts (i.e., this participant had zero red pixels for both positioning concepts). Regarding these three paired measurements, no common features can be recognized: they are from three different participants and three different positions (Fig. 4).

Comparing the three positions (supine, 30° and 90° side lying) for both positioning concepts, 90° side lying lead to the largest total support surface. For CON, the total surface was smallest for 30° side lying, while for LiN this was the case for the supine position (see Table 1).

The surface with high pressure (red) was larger for CON compared to LiN for all three positions. For CON, 30° side lying lead on average to the largest surface with high pressure, while for LiN this was true for 90° side lying (see Table 1).

## Discussion

This pilot study showed that participants positioned in LiN exerted substantially lower pressure on a measurement mattress compared to the same participants positioned in CON. Specifically, areas with high pressure, which is the main factor for the development of decubitus ulcers, were bigger in all positions for CON compared to LiN.

Specific positions have a higher risk concerning the development of decubitus ulcers than others. For example, 90° side lying leads to higher pressure than 30°side lying [13, 14]. Regarding the total amount of pressure, our data support this observation. Indeed, in both LiN and CON, the total support pressure was highest for 90° side lying (Table 1).

Decubitus risk is influenced by intrinsic factors like infections, exsiccosis, weight, incontinence, age and reduced mobility as well as by extrinsic factors such as skin moisture, lifting und positioning techniques, positioning frequency, mattress systems and body hygiene [15]. The main extrinsic reason for the development of decubitus ulcers is thought to be sustained pressure, which leads to poor blood supply of the tissue. The most important factors in order to counteract the pressure are believed to be patients’ movements, pressure distribution and pressure relief. Movement and regular re-positioning from one to another position serve pressure relief. By distributing the pressure, viscoelastic and dynamic mattresses as well as overlays can reduce the risk for decubitus ulcers compared to standard mattresses [16, 17]. It appears that the combination of positioning on soft foam and re-positioning is promising. A re-positioning interval of 4 h with patients being re-positioned on a viscoelastic mattress has been found to be superior to a re-positioning interval of 2 h on a conventional mattress [18].

Positioning does, however, not only serve decubitus prophylaxis. It is also used to positively influence respiratory and therapeutic parameters [19–21]. However, previous studies have usually only investigated the effects on cardiovascular or respiratory parameters or on the development of a decubitus ulcers. Future studies should therefore not only study the pressure distribution on antidecubitus soft foam systems compared to LiN on conventional mattresses. At the same time, parameters such as passive mobility, vital parameters and comfort should be investigated to better understand the positive and negative side effects of positioning methods.

A limitation of this pilot study is that a simple measurement device was used, which is not yet validated. This device can differentiate between high and low pressure, but it does not validly reveal the exact amount of pressure corresponding to each colour (red, yellow and green). Nevertheless, it can be assumed that the randomized approach, which took place within one single forenoon, lead to internally valid data. Using this approach, evidence was found for the hypothesis that LiN leads to a lower measurable total support pressure and to less maximum pressure compared to CON.

In this pilot study, we only investigated healthy participants and no patients. We chose to study two men and two women, one man and one woman being of normal weight and one man and one woman being obese or pre-obese. This was done in order to investigate an arguably average group of individuals. Before this study, a randomized controlled trial had already determined the effects of LiN compared to CON [11]. Dependent variables in that trial were passive motion of the proximal joints, comfort and vital parameters. It was shown that LiN lead to a clinically relevant and significant improvement of the passive range of motion, and that it was perceived as substantially more comfortable than CON. In CON, no changes from pre- to post-positioning could be found [11]. Neither concept had an influence on pulse, blood pressure or respiratory rate [12].

## Conclusion

A previous study had shown an advantage of LiN over CON positioning according to its positive influence on passive range of motion and comfort. The present study confirms that the use of LiN should be preferred over the use of CON due to decreased pressure exerted on the body, a major risk factor for developing pressure sores. A clinical study has to be conducted in order to study the actual effects of LiN compared to CON on the prevention of decubitus ulcers. This controlled trial should aim to study the effects of, at least, the most commonly used positons in clinical practice such as supine, 30° side lying and 90° side lying. The study should answer the question whether applying LiN positions consistently is superior to consistently applying CON positions with regard to the development of decubitus ulcers. As the LiN principles can be applied in all positions, prone and half prone lying or sitting in bed could be investigated as well.

## Abbreviations

CON: Conventional positioning
LiN: Positioning in neutral (Lagerung in Neutralstellung)
